# Duplication within two regions distal to *MECP2*: clinical similarity with* MECP2* duplication syndrome

**DOI:** 10.1186/s12920-023-01465-3

**Published:** 2023-03-06

**Authors:** Keiko Akahoshi, Eiji Nakagawa, Yu-ichi Goto, Ken Inoue

**Affiliations:** 1 Department of Pediatrics, Tokyo Children’s Rehabilitation Hospital, 4-10-1 Gakuen, Musashi-Murayama, Tokyo 208-0011 Japan; 2 Department of Child Neurology, National Center of Neurology and Psychiatry, Tokyo, 187-8551 Japan; 3 Department of Mental Retardation and Birth Defect Research, National Institute of Neuroscience, Tokyo, 187-8502 Japan; 4 Medical Genome Center, National Center of Neurology and Psychiatry, Tokyo, 187-8551 Japan

**Keywords:** Gene dysfunction, *MECP2* duplication syndrome, Progressive neurological disorder, X-linked intellectual disabilities, Xq28 duplication, Chromosomal microarray

## Abstract

**Background:**

X-linked methyl-CpG-binding protein 2 (*MECP2*) duplication syndrome is prevalent in approximately 1% of X-linked intellectual disabilities. Accumulating evidence has suggested that *MECP2* is the causative gene of *MECP2* duplication syndrome. We report a case of a 17-year-old boy with a 1.2 Mb duplication distal to *MECP2* on chromosome Xq28. Although this region does not contain *MECP2*, the clinical features and course of the boy are remarkably similar to those observed in *MECP2* duplication syndrome. Recently, case reports have described duplication in the region distal to, and not containing, *MECP2*. These regions have been classified as the *K/L*-mediated Xq28 duplication region and *int22h1/int22h2*-mediated Xq28 duplication region.

The case reports also described signs similar to those of *MECP2* duplication syndrome. To the best of our knowledge, ours is the first case to include these two regions.

**Case presentation:**

The boy presented with a mild to moderate regressive intellectual disability and progressive neurological disorder. He developed epilepsy at the age of 6 years and underwent a bilateral equinus foot surgery at 14 years of age because of the increasing spasticity in lower extremities since the age of 11. Intracranial findings showed hypoplasia of the corpus callosum, cerebellum, and brain stem; linear hyperintensity in the deep white matter; and decreased white matter capacity. During his childhood, he suffered from recurrent infection. However, genital problems, skin abnormalities and gastrointestinal manifestations (gastroesophageal reflux) were not observed.

**Conclusions:**

Cases in which duplication was observed in the region of Xq28 that does not include *MECP2* also showed symptoms similar to those of *MECP2* duplication syndrome. We compared four pathologies: *MECP2* duplication syndrome with minimal regions, duplication within the two distal regions without *MECP2*, and our case including both regions. Our results suggest that *MECP2* alone may not explain all symptoms of duplication in the distal part of Xq28.

## Background

*MECP2* is an X-linked transcriptional regulator that binds methylated sites in DNA and regulates the expression of a wide range of genes associated with the nervous system [1]. X-linked *MECP2* duplication syndrome, which is prevalent in approximately 1% of X-linked intellectual disabilities, is characterized by infantile hypotonia, severe intellectual disabilities, recurrent infections, progressive spastic disorders, and epilepsy after infancy [2–4]. Collins et al. described the possibility that duplications or gain-of-function mutations in *MECP2* might underlie some cases of X-linked delayed-onset neurobehavioral disorders [5]. In *MECP2* duplication syndrome, different duplication sizes ranging from 0.2–4.0 Mb have been identified [6]. Signs may vary depending on the genes within the region [7], and the role of MECP2 in most of these phenotypes remains unclear.

Duplicated cases in the *K/L-mediated* Xq28 region (R2: region 2) [8–10] and the *int22h1/int22h2-mediated* Xq28 region (R3: region 3) [11–13], which are located distal to *MECP2* and do not contain *MECP2*, have recently been reported. In these case reports, hypotonia, susceptibility to infection, moderate-to-mild intellectual disability, psychiatric signs, and neurological signs were described.

In this report, we describe the case of a 17-year-old Japanese boy with a regressive intellectual disability and progressive neurological disorder. A microarray examination of his DNA identified a 1.2 Mb duplication of maternal origin located 250 kb distal to *MECP2* on chromosome Xq28 and containing the two abovementioned regions. The phenotype of this patient was similar to that of *MECP2* duplication syndrome, although the duplication occurred in a region not containing *MECP2*. We compared clinical manifestations in four regions in male-only cases: cases reported as *MECP2* duplication syndrome with a particularly small region that does not overlap with other regions (R1: region1) [1, 3, 14], the two regions described above (R2 and R3), and our case. We further discussed why they might present similarly despite not containing *MECP*2.

## Case presentation

**Fig. 1:**
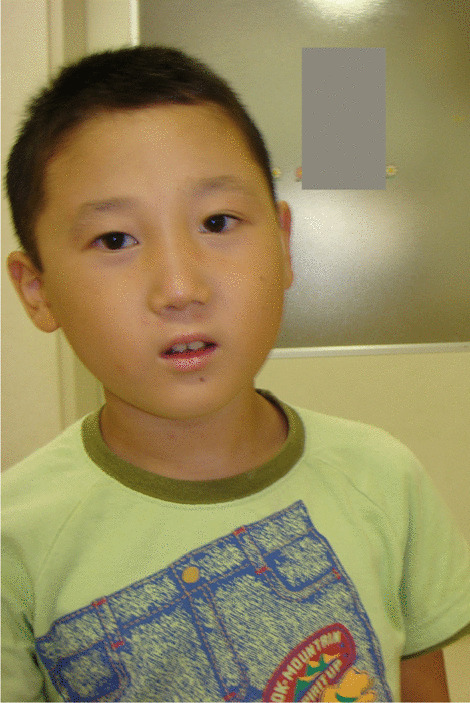
Patient at 7 years and 10 months of age

**Fig. 2:**
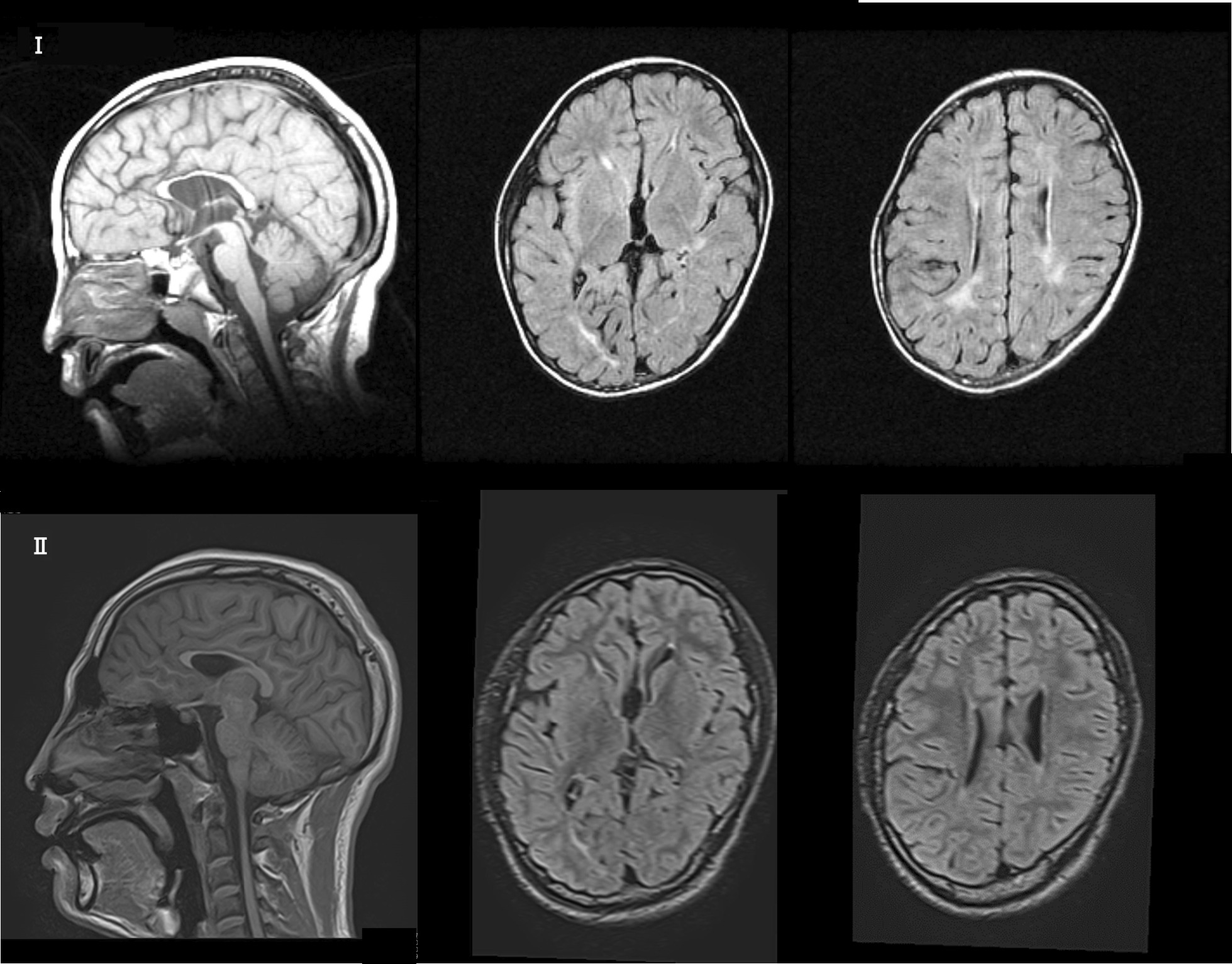
**I** Magnetic resonance imaging (MRI) image of patient at 3 years of age: T1-weighted and FLAIR images exhibit microcephaly; Verga’s cavity; hypoplasia of the corpus callosum, cerebellum, and brain stem; linear hyperintensity in the deep white matter; and unclear hypersensitivity in the cortex and white matter. **II** MRI image at 17 years of age: T1-weighted and FLAIR images show microcephaly; Verga’s cavity; hypoplasia of the corpus callosum, cerebellum, and brain stem; and decreased white matter capacity. The high intensity in the deep white matter observed during infancy was lowered

The boy (Fig. 1) was born at 38 weeks of gestation with an uncomplicated perinatal course and a birth weight of 2868 g. However, his development has been delayed since infancy. The mother of the patient has borderline intellectual functioning, the maternal uncle showed psychomotor disability, and the father and younger brother were healthy. The patient started raising his head at 3 months of age, crawling at 9 months, walking at 21 months, and speaking at 2 years. The patient was susceptible to infections (respiratory infections or otitis media) and often had fevers. On visiting our hospital at the age of 2 years and 4 months, he presented with a small mouth, fair skin, sacral sinus, flattened foot valgus, muscular hypotonia and resulting constipation, and cervical lymph node swelling. However, hypoplastic genitalia, cryptorchidism, and skin abnormalities were not observed. He spoke only three words, and was diagnosed with a psychomotor disability, and received physical, occupational, and speech therapy. Thyroid function and amino acid and organic acid levels were normal, except for mildly decreased serum IgA levels. He could run at the age of 3 years and speak simple sentences at 3.5 years. At 5 years of age, the Wechsler Intelligence Scale for Children^®^ (WISC)-III indicated a mild intellectual disability, with a total IQ of 63. T1-weighted and fluid-attenuated inversion recovery (FLAIR) images of the magnetic resonance imaging (MRI) of his brain exhibited microcephaly; Verga’s cavity; hypoplasia of the corpus callosum, cerebellum, and brain stem; linear hyperintensity in the deep white matter; and unclear hyperintensity in the cortex and white matter (Fig. 2I).

**Fig. 3:**
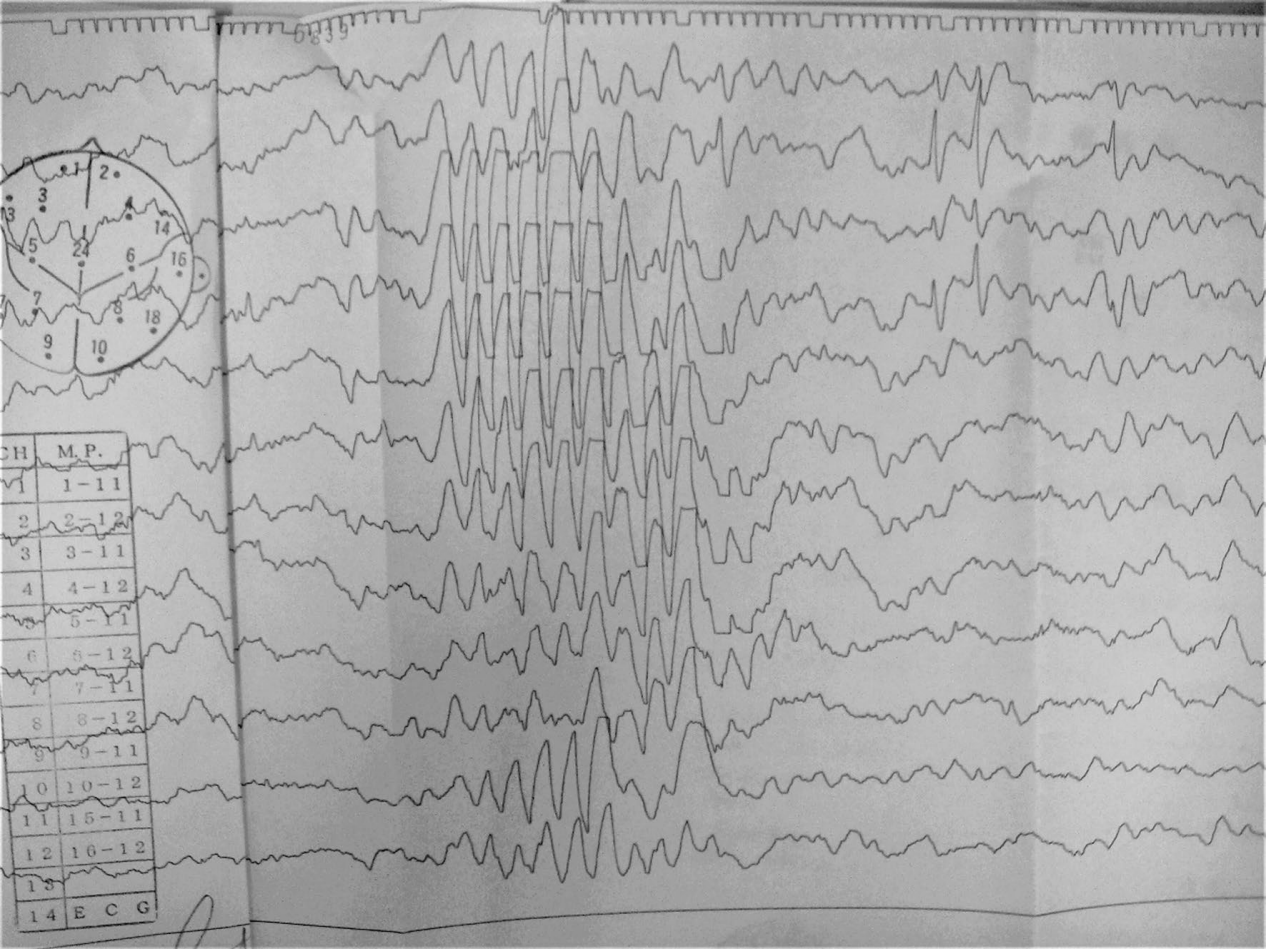
Electroencephalogram findings at the age of 6 years revealed a Θ burst and spike-and-wave connection. (Rt > Lt) in the frontal area of the brain

He developed epilepsy at the age of 6 years and presented with hyperactivity and aggressive behaviors (tantrums and hitting others). Treatment included antiepileptic and psychotropic drugs, such as zonisamide and risperidone, respectively, which helped control epileptic seizures. An electroencephalogram indicated a Θ burst and spike-and-wave connection (lateral view [Rt] > posterior view [Lt]) in the frontal area of the brain (Fig. 3).

At the age of 10 years, the patient persistently exhibited hyperactivity and aggression, in addition to runaway urge, pain insensitivity, and incontinence; furthermore, he could no longer perform the calculations that he could do previously. The patient also presented with moderate intellectual disability.

At 11 years of age, the spasticity of his lower limbs and equinus varus progressed, and he developed difficulty climbing stairs. Consequently, the patient had undergone bilateral equinus foot surgery at 14 years of age, which enabled him to climb stairs. The fifth edition of the Tanaka-Binet Intelligence Scale suggested moderate intellectual disability, with a total IQ of 39 at 16 years of age. In addition to the previous findings, at 17 years of age, brain MRI findings and T1-weighted and FLAIR images revealed decreased white matter capacity, and the high intensities in this area observed during infancy were lowered (Fig. 2II). Currently, he is enrolled in a special-needs school.

### Cytogenetic examinations

**Fig. 4:**
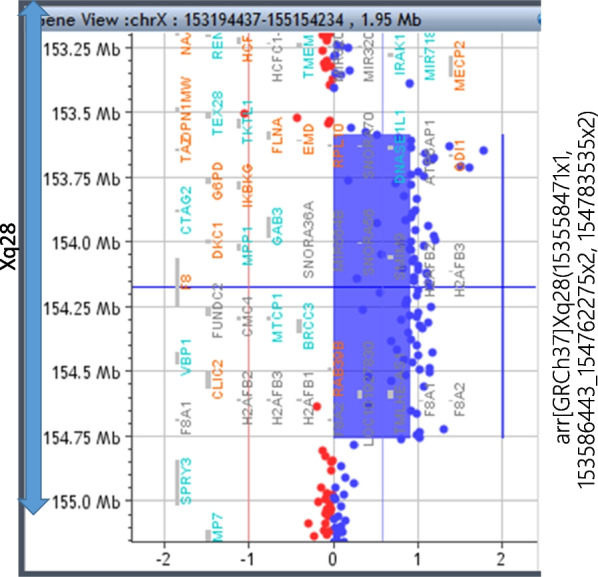
Chromosomal microarray testing: arr[hg19] chrX(153,586,397–154,762,275)X2. (q28, gain, 1.2 Mb)

Conventional chromosomal G-banding on lymphocytes revealed normal 46, XY karyotype. The FMR1 DNA test results were normal. Subtelomeric rearrangements were excluded using telomere fluorescence in situ hybridization assay (using ToTelVysion Probes, Abbott Molecular Inc., IL 60,007 US). Chromosomal microarray testing using the Agilent 60 K Human Genome Comparative Genomic Hybridization Microarray platform (Agilent Technologies,Santa Clara, CA, USA) revealed approximately 1.2 Mb duplication at Xq28, arr[GRCh37]Xq28(153,558,471 × 1, 153586443_154762275 × 2, 154,783,535 × 2) (Fig. 4), which was inherited from his mother.

## Discussion and conclusions

**Fig. 5:**
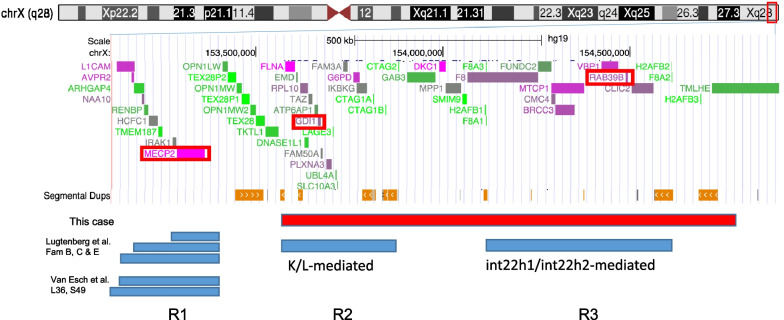
Schematic representation of Xq28, showing the regions of MECP2 duplication syndrome with the minimal regions (R1) and duplication syndromes located distal to MECP2 (R2, R3), and our case

In the distal Xq28 region, duplication frequently occurs because of the high guanine-cytosine content and low copy repeats [15]. Based on a series of studies reporting patients with genomic duplications within this region, multiple genes/regions are likely responsible for the clinical phenotypes observed in patients harboring different segments of duplication. We compared the clinical manifestations of the four regions: R1, R2, R3, and our case involving both R2 and R3 (Fig. 5; Table 1). The 0.3 Mb R2, 0.5 Mb R3, and our case were distal to *MECP2* and did not contain *MECP2*. The causative gene of our case was within these regions and was presumed to be other than *MECP2*.

**Table 1:** Comparison of clinical features of male patients with Xq28 duplication syndrome

			Age	Spasticity of lower legs	Epilepsy	Cranial and brain abnormality	Recurrent -infection	Intellectual disability	Behavioral and autistic problems
*A. Clinical features of male patients with MECP2 duplication syndrome (Region 1)*									
Am J Hum Genet. 2005	Van Esch et al.	L36		4/4	3/4	1/4 microcephaly	3/6	6/6 severe	ND
		S49 III-2	12 years	(+)	(+)	(−)	(+)	severe	(±)
Eur J Hum Genet. 2009	Lugtenberg, et al.	FB II -7	15 years	(+)	(+)	(+)	(−)	mod–severe	(+)
		FB III -1	18 years	(−)	(+)	(+)	(−)	mod	(−)
		FB III -2	10 years	(−)	(+)	(+)	(−)	severe	ND
		FB III -5	6 years	(−)	(−)	ND	(−)	severe	ND
		FC II-I	23 years	(+)	(+)	(+) macrocephaly	(+)	mod–severe	ND
		FC II-II	23 years	(−)	(−)	(+) macrocephaly	(−)	mod–severe	ND
		FE II-1	11 years	(−)	(+)	(−)	ND	mod–severe	ND
		FE II-2	8 years	(−)	(−)	(−)	(−)	severe	ND
Brain Dev. 2013	Shimada et al.	P 3	5 years	(−)	(+)	(+)	ND	severe	ND

			Age	Spasticity of lower legs	Epilepsy	Cranial and brain abnormality	Recurrent infection	Intellectual disability	Behavioral and autistic problems
*B. Clinical features of male patients with K/L-mediated Xq28 duplication syndrome (Region 2)*									
Am J Hum Genet. 2009	Vandewalle et al.	F1 IV.2	6 years	(+) pyramidal sign	ND	(+)	ND	mod IQ50	(−)
		F1 III.10	37 years	(+) pyramidal sign	ND	ND	ND	mod	(−)
		F1 III.19	adult	(±)	ND	ND	ND	mod	(−)
		F2 II.1	3.5 years	ND	(+)	(+) microcephaly	(+)	severe	ND
		F2 II.2	19 months	ND	(−)	(+)	ND		ND
		F3 II.2	7 years	(−)	neonatal convulsion	(+)	ND	mild IQ 58	(−)
		F4 II.1	2 years	ND	(−)	(−)	ND	mild	ND
Am J Med Genet A. 2018	Ward et al.	F1-P1	2 years	(+)	(+)	(+)	(+)		ND
		F2-P4	18 years	ND	(−)	(+)	ND	mild	(−)
		F2-P7	8 years	ND	ND	(+) brachycephaly	ND	mild	(+)
Clincal Genet. 2019	Sinibald et al.	P1	40 years	(±) toes,camptodactyly	(+)	(+) microcephaly	ND	mod	ND
		P2	6 years	ND	ND	(+) microcephaly	ND	mild	ND
		P3	11 years	(+)	ND	(−)	ND	mild	(−) LD
		P4	5 years	ND	ND	(+) microcephaly	ND	mod	ND
		P5	4 years	ND	EEG abnormal	(+) microcephaly	ND	mild IQ67	ND

			Age	Spasticity of lower legs	Epilepsy	Cranial and brain abnormality	Recurrent infection	Intellectual disability	Behavioral and autistic problems
*C. Clinical features of male patients with Int22h-1/int22h-2-mediated Xq28 duplication syndrome (Region 3)*									
J Med Genet. 2011	El-Hattab et al.	F1-1	11 years	(±)	ND	(−)	(+)	(+)	(+)
		F1-2	3 years	ND	ND	ND	(+)	(+)	ND
		F2	3 years	ND	ND	(+) microcephaly	(+)	mild	(+)
		F3	15 years	ND	ND	(−)	(+)	mild IQ57	(+)
Hum. Mutat. 2014	Vanmarsenille et al.	II-1	6.5 years	ND	ND	ND	ND	mod	(+)
		II-2	3 years	ND	ND	ND	ND	mild	ND
		AV1	22 years	ND	ND	(−)	ND	mild IQ61	(+)
		KM1	6.5 years	(+)	(−)	(−)	(+)	mild IQ60	ND
BMC Med Genet. 2015	El-Hattab et al.	F1-1	9 months	ND	ND	ND	(+)	(+)	ND
		F2-1	9 years	ND	ND	ND	(+)	(+)	(+)
		F2-2	6 years	ND	ND	ND	(+)	(+)	(+)
		F3-1	15 years	ND	ND	(+) macrocephaly	(+)	(+)	(+)
		F4-1	12 years	ND	ND	ND	(+)	mild IQ59	(+)

*mod* Moderate, *ND* Not detected, *LD* Learning disability

*K/L-mediated* Xq28 duplication involves 18 genes of which *RPL10, ATP6AP1*, and *GDI1* are highly expressed in the brain, with *GDI1* being the most likely causative gene [8, 15]. *GDI1* has been identified as X-linked intellectual developmental disorder-41 (XLID41). The increased copy number of*GDI1* correlates with severe neurological signs [7, 9]. The region between *int22h1 and int22h2* of Xq28 contains *FUNDC2*, *MTCP1, BRCC3, VBP1, RAB39B, CLIC2,* and some genes of *F8*. The cognitive and neurobehavioral manifestations seen in individuals with *MECP2* duplication syndrome are speculated to result from increased dosages of *CLIC2* and *RAB39B* [13]. *RAB39B* has been identified as X-linked intellectual developmental disorder-72 (XLID72). *RAB39B* is strongly expressed in the brain and encodes a small GTPase that regulates neuronal development [16]. Vanmarsenille et al. reported that increased doses of *RAB39B* affects neuronal development and results in a distinct XLID syndrome characterized by autism and cognitive impairment in men [12].

Our patient exhibited spastic diplegia, intellectual disability, post-infant epilepsy, increased susceptibility to infection, and behavioral problems. The spastic neurological symptoms and intellectual regression were progressive. Comparing the symptoms observed in our case with those in men with the other three duplication syndromes led to the following observations:

First, spastic paralysis of both lower limbs starts around puberty, causing difficulty in climbing stairs and requiring surgical intervention. In R2 duplications, 6 of 15 cases (Vandewalle et al. reported three of seven cases [8], Ward et al. reported one of three cases [9], and Sinibald et al. reported two of five cases [10]) exhibited pyramidal tract signs or spasticity (including a description of toe walking that required heel cord lengthening). R2 contains *GDI1*. It has also been reported that the higher the copy number, the stronger these neurological symptoms, suggesting that *GDI1* is the dosage-sensitive gene responsible for these phenotypes [8, 9]. In R3 duplications, only 1 out of 13 case reports exhibited spasticity [12]. In R1 duplications involving *MECP2*, 7 of 14 cases were reported. Therefore, spasticity occurs more frequently in the duplication of R1 and R2. These areas did not overlap, which may be attributed to the different genetic etiologies in each region. However, progressive spasticity often appears after school age and may not be evident during childhood.

Second, epilepsy can also occur: 3 of the 15 cases with R2 duplications exhibited epileptic phenotypes, whereas none of the 13 cases with R3 duplications had them. In R1 duplications, 10 of the 14 cases reportedly demonstrated epileptic phenotypes. Ward et al. reported that *GDI1* mRNA expression in R2 positively correlates with the severity of intellectual deficits [9]. The likelihood of developing epilepsy is positively correlated with the severity of intellectual deficiency. Cases with R1 duplication presented with severe and intractable epilepsy signs [3, 17], whereas cases with R2 duplication often exhibit relatively mild signs and respond well to medication, as seen in our case.

Third, abnormalities in intracranial findings of the head, including microcephaly, are as follows: In R2 duplications, 11 of 15 cases (Vandewalle et al. reported four of seven cases [8], Ward et al. reported three of three cases [9], and Sinibald et al. reported four of five cases [10]) presented with corpus callosum hypoplasia, and brainstem and cerebellar vermis hypoplasia with ventricular dilatation, suggesting that R2 duplications may cause dysgenesis of the brain during the embryonic period [18]. Yamamoto et al. [7] and Honda et al. [19] stated that duplication of the *GDI1* region is associated with hypoplasia of the corpus callosum in patients. In contrast, only 2 (microcephaly and macrocephaly) of 13 cases of R3 duplications has been reported [11–13]. In cases with R1 duplications, moderate cerebral atrophy with a hypersignal of the white matter in the posterior parts of the cerebral hemisphere was associated with moderate superior vermis atrophy, moderate cortical atrophy, and a hypersignal of the posterior part of the cerebral white matter. In our case, we observed *GDI1*-associated findings, such as corpus callosum, brainstem, and cerebellar vermis hypoplasia and abnormally increased hyperintensity of the posterior periventricular cerebral white matter, that are seen in *MECP*2 syndrome [20, 21].

Fourth, susceptibility to recurrent infections is a unique non-neuropsychiatric feature observed in duplications on chromosome X [22, 23]. In R2 duplications, only 2 of 15 cases exhibited this phenotype. In contrast, recurrent infections were reported in 10 of 13 cases with R3 duplications and 5 of 16 cases with R1 duplications. Therefore, genes within the R1 and R3 regions may be involved in, infection susceptibility. Notably, this phenotype can be overlooked or disregarded during neuropsychiatric evaluation; thus, certain cases may not have been examined in some studies. However, some studies have described associations with genes such as *MECP2, IRAK1*, and *IKBKG* within this region [3, 24], although this remains unclear.

Fifth, delayed psychomotor development and intellectual disability are the main features of Xq28 duplication syndrome and were observed in all cases with increased copy numbers in all three regions. *MECP2* duplication in R1 was associated with more severe intellectual disability; in R2, there were more moderate cases; and in R3, there were more cases with mild to moderate intellectual disabilities. This suggests that the copy number gain of all three regions causes intellectual disability, with *MECP2* effectively having the strongest syndrome, exhibiting even more severe intellectual disability [23]. In contrast, behavioral and neurodevelopmental disorders, including autistic disorders, are not commonly present in patients with duplications in all regions. Neurobehavioral signs, such as attention deficit hyperactivity disorder, impulsivity, and autism spectrum disorder, were present in 9 of 13 cases in the R3 duplicate, whereas only 1 of 15 cases in the R2 duplicate reported behavioral disorders. In R1 duplications, aggressive behavior was reported in only 1 of 13 cases (In R2, F3II.2 by Vandewalle et al. [9] is described as hyperactivity, but this case is excluded because of a perinatal disorder). Thus, neurodevelopmental disorders are particularly common in the *int22h1/int22h2-mediated* Xq28 duplication syndromes. Increasing *RAB39B* doses in this region inhibits neuronal development and has been reported to elicit cognitive dysfunction and behavioral problems in patients with a repetitive copy number increase [12, 25]. Mignogna et al. described, using a *Rab39b-* knockdown mouse model, that the downregulation of *RAB39B* led to increased GluA2 lacking Ca2 + -permeable aminomethylphosphonic acid receptor composition at the hippocampal neuronal surface and increased dendritic spine density that remained in an immature filopodia-like state [26]. These phenotypes affect behavioral performance in a disease-specific manner. Therefore, we speculate that the most likely causative gene for neurodevelopmental disorders in this region is *RAB39B*.

In summary, signs such as spasticity of the lower extremities, epilepsy, and brain development failure may involve genes close to *MECP2,* and behavioral disorders may involve genes in the distal region. Regarding intellectual disabilities, dysfunction in the region containing *MECP2* leads to severe cases, whereas dysfunction in the distal region without *MECP2* presents mild-to-moderate intellectual disability (Table 2). Since the cases of *MECP2* duplication syndrome reported so far vary in size, and many include *GDI1* and/or *RAB39B*, the gene responsible for each symptom remains unclear [27]. In the present study, we confirmed each symptom by comparing it to *MECP2* duplication cases that did not include *GDI1* or *RAB39B*. Although each symptom is relatively mild, our case is highly similar to that of the *MECP2* syndrome.

**Table 2:** Comparison of clinical symptoms in Xq28 duplication syndrome

	MECP2 duplication syndrome	K/L-mediated Xq28 duplication syndrome	Int22h1/Int22h-2-mediated Xq28 duplication syndrome	Our case
Progressive spasticity of lower legs	7/14	6/15	2/13	(+)
Epilepsy	10/14	4/15	0/13	(+)
Cranial and brain abnormality	7/14	11/15	2/13	(+)
Recurrent infection	5/14	2/15	10/13	(+)
Development delay	14/14	15/15	13/13	(+)
Intellectual disability	Severe	Mild–moderate	Mild–moderate	Mild–moderate
Behavioral and autistic problems	2/14	1/15	9/13	(+)

We speculated that the causative gene for each sign may not be *MECP2* alone. The similarity between the symptoms observed in our case and those in *MECP2* duplication syndrome may be because *MECP2* regulates the functions of other genes in Xq [4]. Therefore, *MECP2* dysfunction can impair the expression of other genes, and the same signs are caused by the dysfunction of each gene. Therefore, the same symptoms may occur due to the dysfunction of different genes. Further studies are necessary to confirm this hypothesis.

## Data Availability

The datasets used in the current study are available from the corresponding author upon reasonable request.

## Abbreviations

*MECP2*: X-linked methyl-CpG-binding protein 2
IgA: Immunoglobulin A
*FLAIR*: Fluid-attenuated inversion recovery
MRI: Magnetic resonance imaging
IQ: Intelligence quotient
Rt: Right
Lt: Left
*FMR1*: Fragile X mental retardation1
*RPL10*: Ribosomal protein L10
*ATP6AP1*: ATPase H + transporting accessory protein 1
*GDI1*: GDP dissociation inhibitor 1
*FUNDC2*: FUN14 domain containing 2
*MTCP1*: Mature T cell proliferation 1
*BRCC3*: BRCA1/BRCA2-containing complex subunit 3
*VBP1*: VHL binding protein 1
*RAB39B*: Member RAS oncogene family
*CLIC2*: Chloride intracellular channel 2
*F8*: Coagulation factor VIII
*IRAK1*: Interleukin 1 receptor associated kinase 1
*IKBKG*: Inhibitor of nuclear factor kappa B kinase regulatory subunit gamma
